# High-activity Classical and Alternative Complement Pathway Genotypes—Association With Donor-specific Antibody-triggered Injury and Renal Allograft Survival

**DOI:** 10.1097/TXD.0000000000000978

**Published:** 2020-02-10

**Authors:** Blanka Mező, Roman Reindl-Schwaighofer, Farsad Eskandary, Andreas Heinzel, Markus Wahrmann, Konstantin Doberer, Andreas Heilos, Gregor Bond, Johannes Kläger, Nicolas Kozakowski, Helmuth Haslacher, Rainer Oberbauer, Ondřej Viklický, Petra Hrubá, Philip F. Halloran, Krisztina Rusai, Zoltán Prohászka, Georg A. Böhmig

**Affiliations:** 1 Research Laboratory, IIIrd Department of Internal Medicine and MTA-SE Research Group of Immunology and Hematology, Hungarian Academy of Sciences and Semmelweis University, Budapest, Hungary.; 2 Division of Nephrology and Dialysis, Department of Medicine III, Medical University of Vienna, Vienna, Austria.; 3 Department of Pediatrics and Adolescent Medicine, Medical University of Vienna, Vienna, Austria.; 4 Department of Pathology, Medical University of Vienna, Vienna, Austria.; 5 Department of Laboratory Medicine, Medical University of Vienna, Vienna, Austria.; 6 Department of Nephrology, Transplant Center, Institute for Clinical and Experimental Medicine, Prague, Czech Republic.; 7 Alberta Transplant Applied Genomics Centre, ATAGC, University of Alberta, Edmonton, AB, Canada.

## Abstract

**Background.:**

Complement may contribute to donor-specific antibody (DSA)-triggered transplant injury. Here, we investigated whether the intrinsic strength of classical pathway and alternative pathway (AP) relates to the pathogenicity of DSA.

**Methods.:**

Classical pathway and AP high-activity genotypes were defined according to C4 gene copy number and the presence of functional polymorphisms in C3 (C3_102G_), factor B (fB_32R_), and factor H (fH_62V_) genes. Associations of these genotypes with blood complement profiles and morphologic/molecular rejection features were evaluated in a cohort of 83 DSA-positive patients (antibody-mediated rejection [AMR], n = 47) identified upon cross-sectional screening of 741 kidney allograft recipients ≥180 days posttransplantation. Associations with long-term graft survival were evaluated in a larger kidney transplant cohort (n = 660) not enriched for a specific type of rejection.

**Results.:**

In the cohort of DSA-positive subjects, the number of C4 gene copies was related to C4 protein levels in serum and capillary C4d staining, but not AMR activity. Patients with a high-activity AP complotype, which was associated with complement consumption in serum, showed enhanced microcirculation inflammation (median glomerulitis plus peritubular capillaritis score, 2 [interquartile range, 0–4 versus 1 0–2]; *P* = 0.037). In the larger transplant cohort, this complotype was associated with a slightly increased risk of graft loss (hazard ratio, 1.52; 95% confidence interval, 1.02-2.25; *P* = 0.038 and multivariable Cox model, 1.55; 1.04-2.32; *P* = 0.031).

**Conclusions.:**

Our study suggests a contribution of complement genetics to the phenotypic presentation of AMR. Future studies will have to clarify whether a possible association of AP strength with graft survival relates to enhanced antibody-triggered injury.

## Abstract 2

Supplemental Digital Content is available in the text.

Complement is well established to play a multifaceted role in organ transplantation, including a potential contribution to the pathogenesis of antibody-mediated rejection (AMR).^1,2^ Considering a role of the classical pathway (CP) of complement as a trigger of donor-specific antibody (DSA)-triggered inflammation in the microvasculature, one would expect that its genetic background would determine the severity of rejection.

One determinant of CP activity may be a substantial gene copy number variation (CNV) of key component C4.^3^ Large cohort studies evaluating associations of C4 CNV with long-term renal transplant survival have revealed controversial results.^4,5^ Granular endpoints, such as the development of AMR or its phenotypic presentation, however, have not been evaluated. In addition, one may argue that the extent of DSA-triggered complement activation depends on the presence or absence of functional single nucleotide polymorphisms (SNPs) determining the strength of the alternative pathway (AP) amplification loop, which is critical for full CP activation.^6^ An interesting approach in this context may be the definition of “high-activity” complotypes, based on a combination of different functional SNPs determining the activity of the AP convertase.^7^ This concept may be supported by experimental models, including in vitro add-back assays, where specific variants of C3 (C3_102G_, confers resistance toward regulation), factor B (fB_32R_ forms AP convertase more efficiently), and factor H (fH_62V_ binds C3 less strongly and is a worse cofactor for factor I) conferred increased activity of the AP convertase, yielding 6-fold higher hemolytic activity compared with “protective” variants C3_102R_, fB_32Q_, and fH_62I_.^8^

Based on the presumption that activation of the complement cascade contributes to microcirculation inflammation in AMR, we hypothesized that gene variants reflecting high-activity of the CP and/or AP determine the extent of DSA-triggered allograft injury. Our present study included (1) an analysis of genetic variants associated with complement activity in a specific cohort of 83 DSA-positive kidney transplant patients subjected to allograft biopsies^9^ and (2) a subsequent analysis of the clinical impact of a high-activity AP complotype in a large kidney transplant cohort not enriched for a specific type of rejection.^10^

## MATERIALS AND METHODS

### Study Design and Patients

**FIGURE 1.**
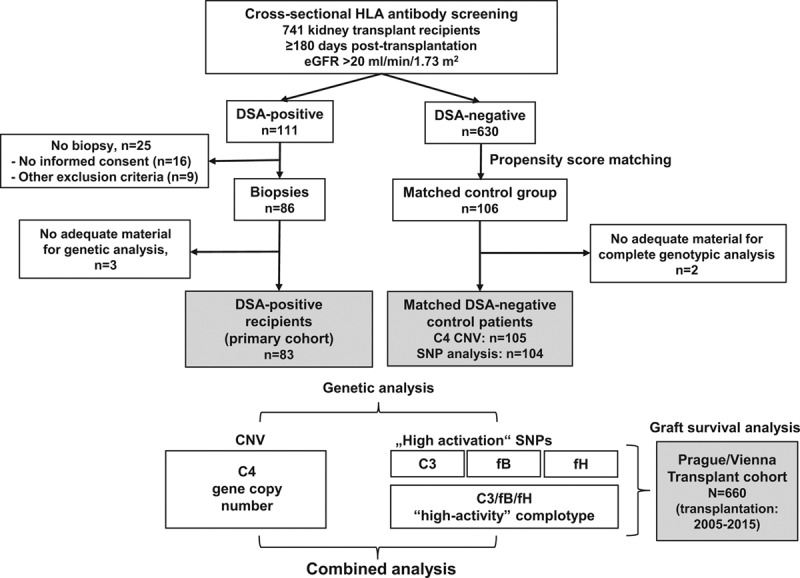
Study flow chart. Systematic cross-sectional antibody-mediated rejection (AMR) screening of a cohort of 741 kidney transplant recipients led to the identification of 111 donor-specific antibody (DSA)-positive recipients. Eighty-six DSA-positive patients underwent protocol biopsies. For 83 subjects (primary study cohort), adequate material for genotyping was available. A control group of 106 recipients was defined by propensity score matching as described in the methods section. A large prospective bicenter kidney transplant cohort (Prague/Vienna cohort; 660 transplants) was used to evaluate associations of genotyping results with long-term allograft survival. CNV, copy number variation; eGFR, estimated glomerular filtration rate; fB, factor B; fH, factor H; SNP, single nucleotide polymorphism.

The study included a primary cohort of 83 kidney transplant recipients (transplantation between 1990 and 2014) who all underwent allograft biopsies for a positive DSA test result (Tables 1 and 2). As shown in Figure 1, patients were identified upon prospective cross-sectional HLA antibody screening of a cohort of 741 recipients (screening period: October 2013 through February 2015), within an interventional trial designed to evaluate the effect of bortezomib in late AMR (BORTEJECT trial; www.clinicaltrials.org: NCT01873157; registration in June 7, 2013).^9,11^ The protocol of the trial has earlier been described in detail.^9,11^ Key inclusion criteria were as follows: (1) age >18 years, (2) stable allograft function after ≥180 days posttransplantation, and (3) an estimated glomerular filtration rate >20 mL/min per 1.73 m^2^. Patients with acute graft dysfunction were excluded and, accordingly, all included patients were subclinical in response to newly detected DSA. One-hundred eleven patients were DSA-positive and 86 of these patients were subjected to protocol biopsies, on average, 23 days (median, interquartile range [IQR], 15–42 d) after DSA detection. In all study subjects, biological material was obtained before therapeutic interventions within or outside the BORTEJECT trial. For 83 recipients, adequate material for detailed complement analysis was available. Sera and whole blood samples were collected, processed and stored at the biobank facility of the Medical University of Vienna.^12^ For 41 of the 83 study patients, also results of pretransplant DSA testing were available (pretransplant single antigen bead testing was implemented in our routine in July 2009), and 58% of these subjects were DSA positive already before transplantation (Table 1).

**TABLE 1.**
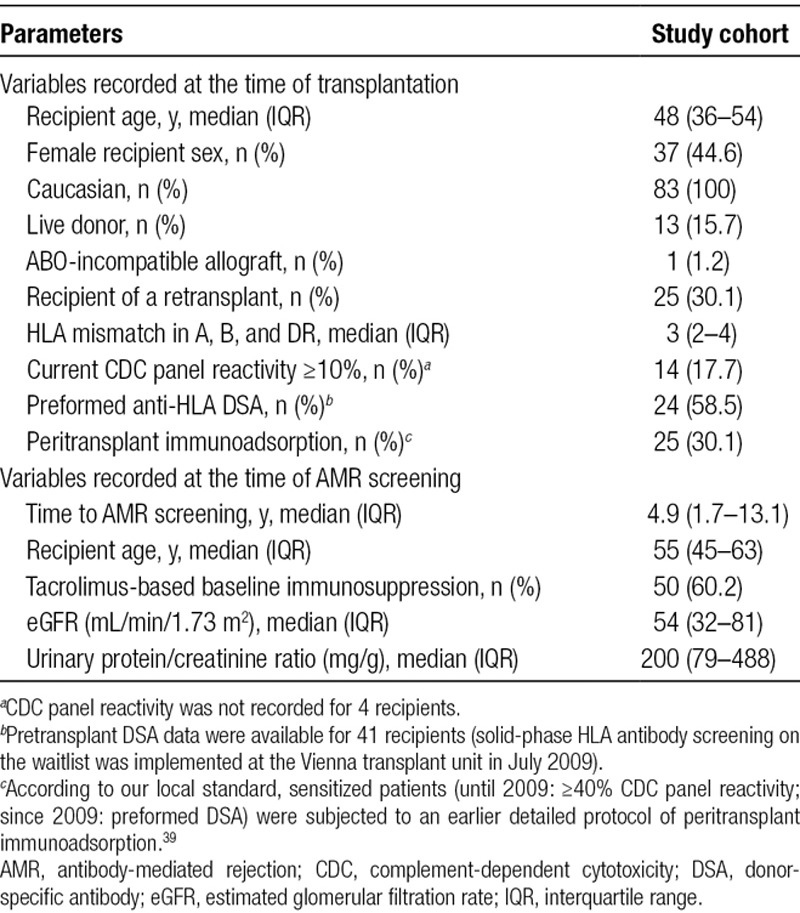
Baseline characteristics—primary study cohort of 83 DSA-positive recipients

For group comparisons in relation to posttransplant DSA status, 106 of the 630 DSA-negative recipients (who according to the trial protocol were not subjected to protocol biopsies) were propensity score-matched with the DSA-positive study patients, following a previously described algorithm.^13^ With the exception of more frequent desensitization among DSA-positive patients, baseline characteristics were not different (**Table S1**, **SDC**, http://links.lww.com/TXD/A243).

The association of a high-activity AP complotype with long-term graft survival was evaluated in 660 renal transplants from the prospective Vienna/Prague kidney transplant cohort (transplantation between 2005 and 2015; **Table S2**, **SDC**, http://links.lww.com/TXD/A243).^10^

The study was approved by the institutional review boards of the Medical University of Vienna (EK267/2011, EK1515/2012) and the Institute for Clinical and Experimental Medicine in Prague (G 05-04-03 and A 13-02-01 [83/13]). The clinical and research activities being reported are consistent with the Principles of the Declaration of Istanbul as outlined in the “Declaration of Istanbul on Organ Trafficking and Transplant Tourism.”

### Biopsies

The work-up of index biopsies performed within the BORTEJECT trial has earlier been described in detail.^9,13^ Rejection-associated lesions were scored following the Banff 2015 scheme.^14^ In parallel, biopsies were subjected to microarray-based gene expression analysis using the Molecular Microscope Diagnostic System.^9,13^

### Antibody Detection

HLA reactivity patterns (mean fluorescence intensity threshold >1000) were analyzed using LABScreen Single Antigen assays (One Lambda; A Thermo Fisher Scientific Brand, Canoga Park, CA) as earlier detailed.^15^ Serum samples were heat-inactivated (30 min, 56°C) to prevent complement interference. Complement fixation was assessed using C1qScreen assays (One Lambda; mean fluorescence intensity threshold >500).

### Genetic Analyses

#### Primary Study Cohort

C4 copy number genotyping was performed using the method based on the TaqMan real-time polymerase chain reaction (PCR) technology.^16^ Primers (Integrated DNA Technologies, Coralville, IA) of the *C4* genes and specific fluorescein amidite-labeled fluorogenic probes for *C4A* and *C4B* on 4 of their 5-base pair differences on exon 26 were designed by Primer Express software (**Table S3**, **SDC**, http://links.lww.com/TXD/A243). For determining the quantity of the *C4A* and *C4B* genes, two separate reactions were performed in 3 parallels for each measurement. Both reaction mixtures contained 2x Maxima Probe/ROX qPCR Master Mix (number K0231; Thermo Fisher Scientific), 20x VIC-labeled RNase P detection mix (number 4403326; Thermo Fisher Scientific), forward and reverse primers (6 µmol/L), genomic DNA template, and either C4A- or C4B-specific probes in a total volume of 20 µl. Real-time PCR was performed using the Rotor-Gene Q instrument (QIAGEN, Hilden, Germany) and programmed as follows: the reaction was initiated by an incubation step at 95°C for 10 minutes, followed by 35 cycles of 95°C for 15 seconds and 60°C for 50 seconds, the fluorescence intensity was measured during the step of 60°C. Samples with known *C4* CNV served as controls.

DNA samples were also genotyped for 3 SNPs, 1 in C3 (C3_R102G_, rs2230199), 1 in factor H (fH_V62I_, rs800292), and 1 in factor B (fB_R32Q_, rs641153). Factor H polymorphism was determined by TaqMan SNP real-time PCR assay.^17^ C3 and fB SNPs were determined by restriction fragment length polymorphism assay. The amplicons were subsequently subjected to restrict digestion with HhaI (ER1851; Thermo Fisher Scientific, Waltham, MA) (C3_R102G_) and MspI (number R0106L; New England Biolabs) (fB_R32Q_) enzymes. DNA-sequencing was also used to validate the fB SNP assay and also for selected cases to confirm the results. Primer sequences and PCR conditions are provided in **Table S4**, **SDC**, http://links.lww.com/TXD/A243.

#### Vienna/Prague Kidney Transplant Cohort

Donor and recipient DNA was genotyped using the iGeneTRAiN transplant array.^18^ The rs2230199 variant was directly covered on the genome-wide association study array. The rs800292 and rs641153 variants were imputed using the 1000 Genomes Project Phase 3 and Genome of the Netherlands v5 as reference panels.^19-21^ High-resolution HLA types for HLA-A, B, C, DPB1/DPA1, DQB1/DQA1, and DRB1 were imputed using SNP2HLA v1.0, with the type 1 Diabetes Genetics Consortium as a reference panel.^22^ HLA eplet mismatch scores were calculated on the basis of the imputed high-resolution HLA genotypes using HLAMatchmaker.^23^

### Detection of Complement Components in Peripheral Blood

As earlier described in detail,^13^ serum concentrations of C3 (reference range, 0.9–1.8 g/L) and C4 (reference range, 0.15–0.55 g/L) were assessed by turbidimetry (Beckman Coulter, Brea, CA). Total serum complement activity of the classical pathway (50% hemolytic complement activity [CH50] assay) was determined using an in-house hemolytic-titration assay based on Mayer’s method (reference range, 48–103 U/mL).^24^ Complement AP activity (reference range, 70%–130%) was determined by a commercial ELISA kit (Wieslab, Eurodiagnostica, Malmö, Sweden).

### Statistical Analysis

Continuous data were expressed as the median and IQR, and categorical variables as absolute and relative frequencies. Fisher exact or chi-square tests were used to compare categorical data, and Mann-Whitney *U* or Kruskal-Wallis tests for comparison of continuous data. As earlier described in detail,^13^ a matched DSA-negative control group was defined using propensity score matching (selected variables: female sex, recipient age at transplantation, number of prior transplantations, HLA mismatch, cytotoxic panel reactivity, and protein/creatinine ratio). Kaplan-Meier analysis was applied for calculation of survival. Mantel-Cox log-rank test was used for comparison of survival between strata. Associations between AP complotype and graft survival in the Prague/Vienna cohort was evaluated using a multivariable Cox proportional hazards model adjusted for recipient age >65 years (threshold for allocation in the Eurotransplant senior program), donor age and sex, underlying renal disease (glomerulonephritis versus other diseases), donor type (living versus deceased donor), HLA eplet mismatch, retransplantation, tacrolimus-based maintenance immunosuppression, and induction therapy with a depleting antibody. A 2-sided *P* < 0.05 was considered statistically significant. For statistical analysis, IBM SPSS Statistics 24 (IBM Corporation, Armonk, NY) was used.

## RESULTS

Baseline characteristics of the primary study cohort of 83 DSA-positive renal allograft recipients are provided in Table 1. Forty-seven study patients were diagnosed with AMR (C4d deposition in 21 cases), the majority (n = 32) showing a chronic active phenotype (Table 2).

**TABLE 2.**
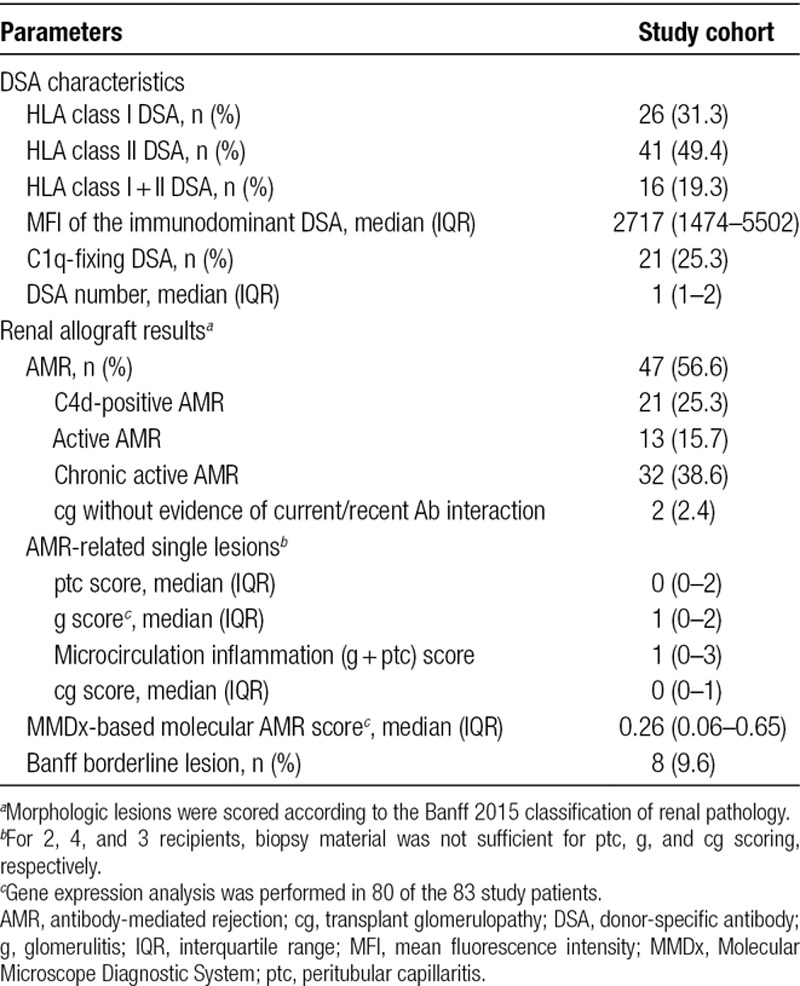
Immunologic data and biopsy results—primary study cohort of 83 DSA-positive recipients

### C4 Gene Copy Number in DSA-positive Recipients

**FIGURE 2.**
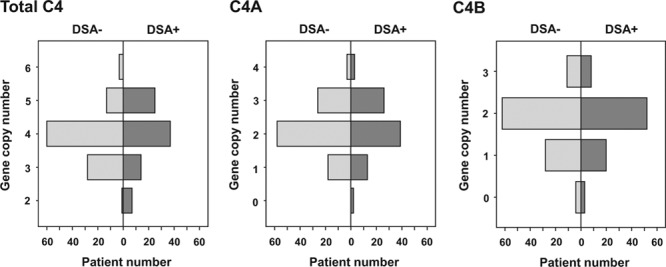
Recipient gene copy number of C4, C4A, and C4B in donor-specific antibody (DSA)-positive study patients and DSA-negative propensity score-matched control subjects.

**FIGURE 3.**
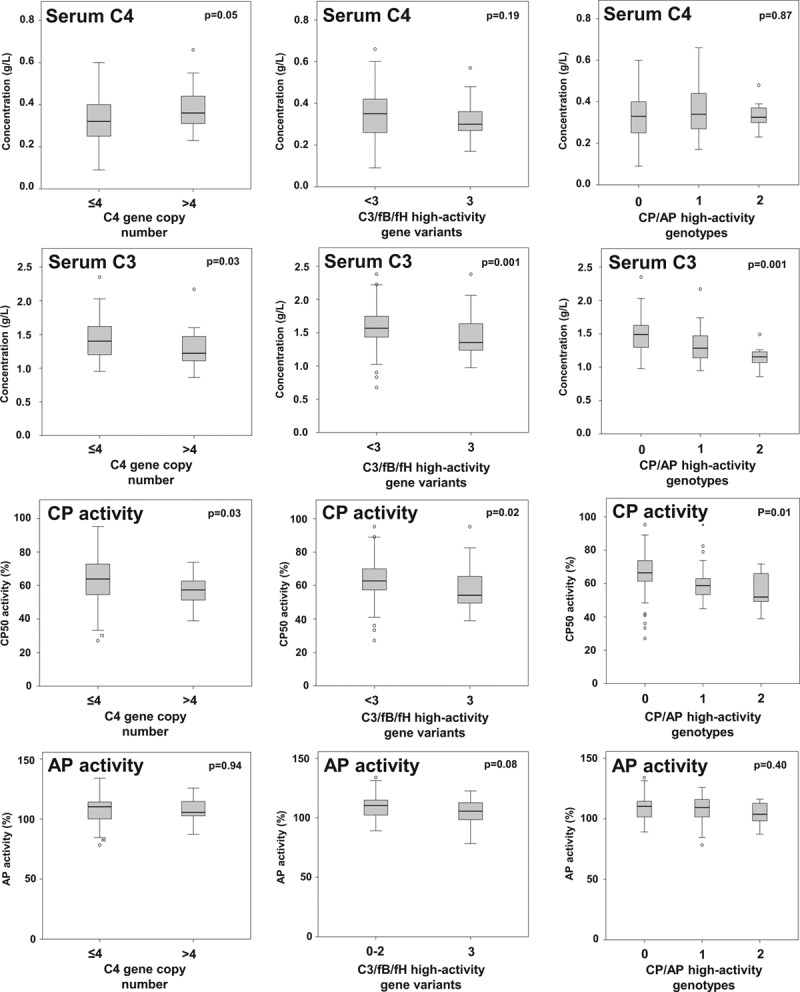
Serum C4 and C3 concentrations and % classical pathway (CP) and alternative pathway (AP) activity in relation to (A) the intrinsic strength of the classical (C4 gene copy number) and (B) alternative pathways (C3/factor B [fB]/factor H [fH] high-activity complotype), and (C) a compound analysis of risk genotypes. Box plots indicate the median, interquartile range, and the minimum and maximum of the measures. Outliers are indicated as circles. Mann-Whitney *U* and Kruskal-Wallis tests were used for group comparison. CH50, 50% hemolytic complement activity.

**FIGURE 4.**
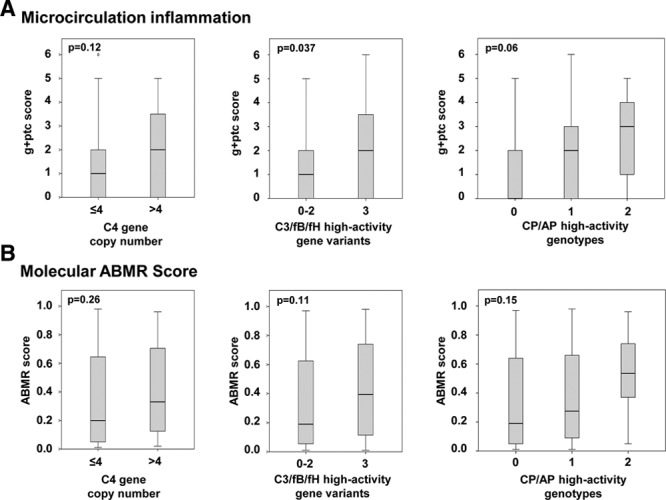
Microcirculation inflammation (g + ptc score) and molecular antibody-mediated rejection (AMR) scores in relation to (A) the intrinsic strength of the classical (C4 gene copy number) and (B) alternative pathways (C3/factor B [fB]/factor H [fH] high-activity complotype), and (C) a compound analysis of high-activity genotypes. Box plots indicate the median, interquartile range, and minimum and maximum of the measures. Outliers are indicated as circles. Mann-Whitney *U* and Kruskal-Wallis tests were used for group comparison. AP, alternative pathway; CP, classical pathway, g, glomerulitis; ptc, peritubular capillaritis.

**FIGURE 5.**
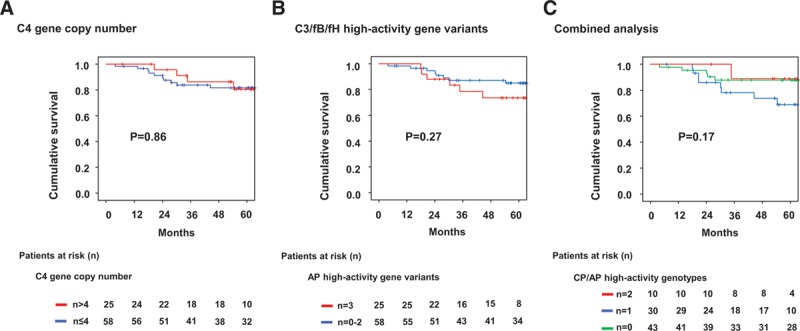
Kaplan-Meier death-censored kidney allograft survival in relation to (A) the intrinsic strength of the classical (CP; C4 gene copy number) and (B) alternative pathways (AP; C3/factor B [fB]/factor H [fH] high-activity complotype), and (C) a combined analysis of high-activity genotypes. The Mantel-Cox log-rank test was used to compare survival rates between groups.

C4, C4A, and C4B copy number distributions were similar between DSA-positive and DSA-negative patients (Figure 2). For statistical comparison, study patients were categorized as having a higher (>4, n = 25) versus lower (≤4, n = 58) C4 gene copy number. A higher copy number was associated with increased C4 protein levels and lower C3 and CH50 levels (Figure 3). There was no significant association with microcirculation inflammation (glomerulitis [g] + peritubular capillaritis [ptc] score: 2 (median [IQR], 0–4) versus 1 [0–2] in patients with ≤4 C4 copies; *P* = 0.12) or molecular activity of AMR (AMR score, 0.20 [IQR, 0.05–0.65] versus 0.33 [0.12–0.72]; *P* = 0.26) (Figure 4). C4d-positive AMR, however, was more frequent among patients with >4 C4 copies (n = 10/25 [40%] versus 11/58 [19%]; *P* = 0.04). Groups showed comparable death-censored 60-month graft survival from the day of index biopsy (81% 5-y graft survival in both strata; univariate Cox model: hazard ratio [HR], 0.90; 95% confidence interval [CI], 0.28-2.87; *P* = 0.86) (Figure 5).

### High-activity C3/fB/fH Complotype in DSA-positive Recipients

The genotypic distributions of C3 (rs2230199; c.304C > G; p.R102G; C3S versus C3F), fB (rs641153; c.95G > A; FB R32Q), and fH (rs800292; c.184G > A; FH V62I), which were not different between DSA-positive and DSA-negative subjects, are detailed in **Table S5**, **SDC**, http://links.lww.com/TXD/A243. Separate analysis of individual variants among DSA-positive subjects revealed lower C3 levels and CP/AP total activity in serum in C3_102G_, and lower levels of CH50 in fH_62V_ individuals, but no significant associations with biopsy results or 5-year survival (**Table S6**, **SDC**, http://links.lww.com/TXD/A243).

The 3 gene variants were then combined to define a high-activity C3/fB/fH complotype, defined by the concomitant presence of at least 1 risk allele in each of the analyzed genes (C3_102G_, fB_32R_, and fH_62V_ variants). As shown in Table 3, 25 patients had a high-activity C3/fB/fH complotype, while 58 patients had only 1 (n = 3) or 2 (n = 55) risk alleles. Analysis of blood complement profile revealed significantly lower C3 and CH50 levels among patients with the high-activity complotype (Figure 3).

**TABLE 3.**
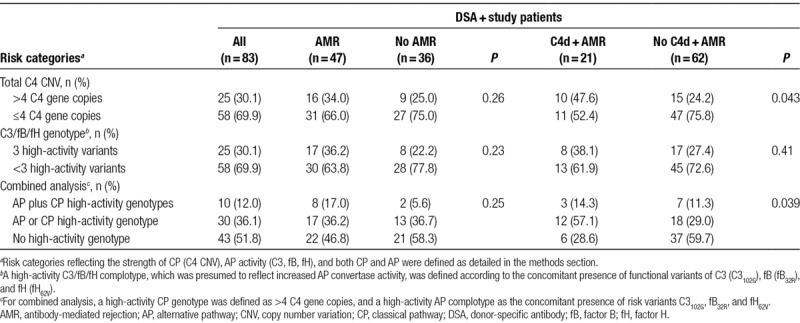
Complement genetic results in relation to biopsy results—primary study cohort of DSA-positive recipients

As shown in Figure 4, the high-activity C3/fB/fH complotype was associated with pronounced microcirculation inflammation (median g + ptc score: 2 [IQR, 0–4] versus 1 [0–2]; *P* = 0.037). Molecular AMR scores were numerically higher, but differences did not achieve statistical significance (median, 0.40; [IQR, 0.11–0.74] versus 0.19 [0.05–0.63]; *P* = 0.11). Seventeen of the 25 patients (68%) with a high-activity complotype were diagnosed with AMR, as compared to 30/58 subjects (52%) without this complotype (*P* = 0.23). Five-year death-censored graft survival from the time of index biopsy was 74% and 85% in patients with and without a high-activity complotype, respectively (*P* = 0.27; univariate Cox model: HR, 1.80 [95% CI, 0.63-5.21], *P* = 0.29) (Figure 5). In a separate analysis of the 106 nonbiopsied DSA-negative matched control subjects, no survival differences were observed (5-y graft survival: 87% versus 90%; *P* = 0.62). Among 19 recipients who never had documented pretransplant and posttransplant DSA only 3 graft losses were recorded, which all occurred in patients without a high-activity AP complotype (data not shown).

A combined analysis of C4 CNV and AP high-activity C3/fB/fH complotype did not considerably affect the results obtained in the separate analyses of CP and AP genotypes. We observed a decrease in C3 and CH50 levels in patients with 1 or 2 high-activity constellations (Figure 3), and, in parallel, a nonsignificant increase in g + ptc and molecular AMR scores (Figure 4). As in the separate analysis of C4 CNV, we found a difference in the rate of C4d-positive AMR (Table 3). Death-censored graft survival was not different between groups (Figure 5).

### High-activity C3/fB/fH Complotype and Graft Survival in a Large Bicenter Cohort of Kidney Transplants

**FIGURE 6.**
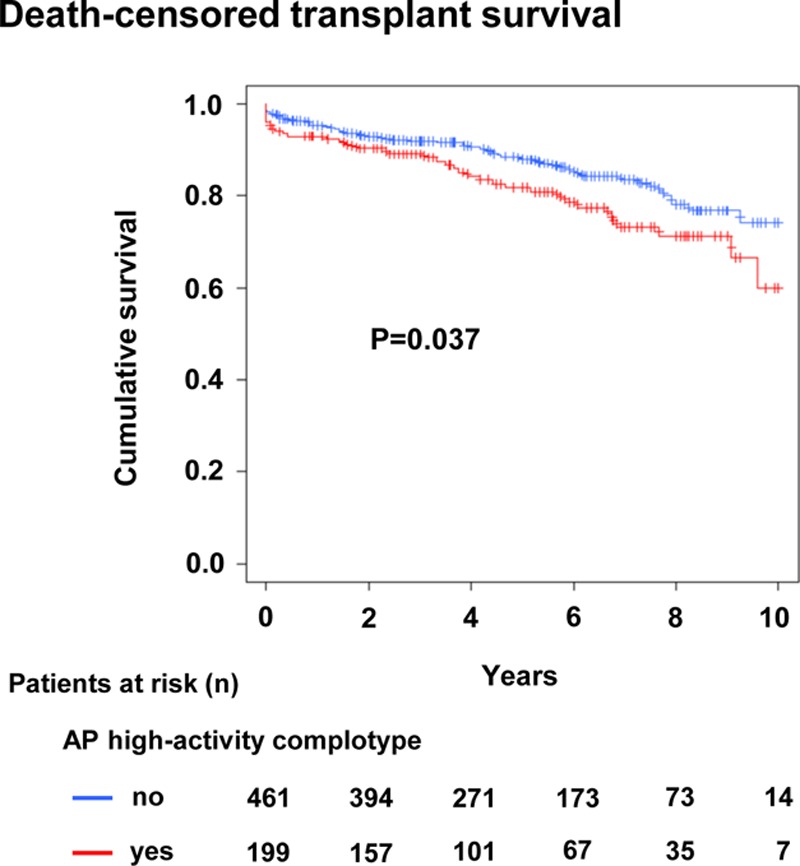
Kaplan-Meier death-censored kidney allograft survival in relation to the alternative pathway (AP) high-activity C3/factor B/factor H complotype at a recipient level in the Vienna/Prague transplant cohort (660 kidney transplants).

Finally, we investigated whether our finding of enhanced microcirculation inflammation in DSA-positive patients with a high-activity C3/fB/fH complotype translates into adverse graft survival in a large prospective cohort of kidney transplant recipients (n = 660; baseline characteristics: **Table S2**, **SDC**, http://links.lww.com/TXD/A243) not enriched for a specific type of rejection. In this cohort, genotype distributions and allele frequencies were similar to the primary study cohort, with the exception of a slight difference in the distribution of C3 genotypes (**Table S7**, **SDC**, http://links.lww.com/TXD/A243). As shown in Figure 6, death-censored graft survival was worse in patients harboring the high-activity complotype (*P* = 0.037), with a significant increase in the risk of graft loss (univariate Cox model: HR, 1.52 [95% CI, 1.02-2.25]; *P* = 0.038). Multivariate analysis adjusted for potential confounders of graft survival (recipient and donor age, recipient and donor sex, underlying renal disease, retransplantation, donor type, HLA eplet mismatch, tacrolimus-based maintenance immunosuppression, and induction therapy with a depleting antibody) showed a HR for graft loss of 1.55 (95% CI, 1.04-2.32; *P* = 0.031) for the high-activity complotype (**Table S8**, **SDC**, http://links.lww.com/TXD/A243).

## DISCUSSION

Our present study suggests that distinct genotypic variants modulating the intrinsic strength of the AP amplification loop may influence the pathogenicity of DSA. A major result obtained in our cohort of DSA-positive recipients was that a high-activity C3/fB/fH complotype (but not C4 CNV) was associated with pronounced inflammation in the transplant microvasculature. In support of potential clinical relevance of assessing the genetic background of the AP, analysis of a larger cohort of 660 kidney transplants revealed that the same complotype was associated with a slight increase in the risk of graft failure. Interpreting our results, we are aware of the preliminary nature of our study. One may speculate that associations between AP strength and antibody-triggered inflammation were related to inherent differences regarding CP amplification via the AP loop. Considering the lack of granular data on DSA and AMR occurrence in our larger cohort, however, the contribution of antibody-triggered complement activation to observed survival differences remains to be established in additional cohorts.

The design of our present study was based on the presumption that (1) full complement activation significantly contributes to microcirculation inflammation triggered by DSA and (2) that the extent of complement activation in this context is not only determined by the type and binding strength of DSA, but also by the intrinsic strength of complement. Full activation of the CP to terminal complement activation may thereby critically depend on the genetic background of a set of different key factors. Given the important role of CP amplification via the AP loop,^6^ these may include also gene variants that determine the activity of the AP (C3bBb) convertase.

Variants of AP components, such as C3, fB and regulatory component fH, are well established to predispose to native kidney disease related to complement dysregulation.^25^ Their impact, however, is less well understood in the context of clinical transplantation. Systematic studies evaluating common functional gene variants of C3 have revealed conflicting results,^26,27^ and, more recently, a broad analysis of 505 tagged SNPs in 47 complement-associated genes has failed to demonstrate any meaningful associations with transplant outcomes.^28^ As the complement cascade is regulated at multiple levels and complement dysregulation may impact on both impaired host defense as well as increased risk for autoimmune injury, a high level of conservation or redundancy in key regulator pathways is fundamental. Most single variants therefore only have a small impact on overall complement activity or may be counterbalanced by other regulators of complement. One may argue that a complotype integrating more than one genetic variant, affecting different levels in the complement cascade may therefore better predict the level of overall complement activity in a single patient. This synergism of different genes associated with increased complement activity at different levels, however, has not yet been systematically studied in transplant patients.

There is experimental evidence that 3 distinct risk variants of key components C3 (C3_102G_), fB (fB_32R_), and fH (fH_62V_) synergize to increase the activity of the AP convertase. Combining these risk variants in add-back assays was shown to yield a 6-fold higher hemolytic activity than protective variants C3_102R_, fB_32Q_, and fH_62I_.^8^ Considering such experimental data, the definition of disease-associated complotypes—based on the combination of risk variants in different genes encoding for key components of the complement cascade—is mechanistically plausible and may represent a useful strategy to define individual risk constellations.^7^

A high C4 gene copy number in our patients was, as in earlier cohorts,^3^ associated with increased C4 protein levels. Even though capillary deposition of C4 split product C4d was more frequent among patients with a high copy number, there was no significant effect on morphologic/molecular AMR activity and survival rates. This may be in line with earlier studies suggesting that C4d deposition per se may not necessarily associate with ongoing rejection,^29^ and recent experimental data have shown that C4d may even dampen immune cell activation.^30^ In a mouse model, C4 deficiency had no effect renal allograft survival,^31^ and in a large cohort of approximately 2000 kidney transplants we found no impact of C4 CNV on long-term graft survival.^4^ Finally, in strong support of a role of CP-independent mechanisms of injury, in a recent interventional trial performed in kidney transplant recipients with late active AMR, targeting C1 subunit C1s to block CP activation led to the elimination of capillary C4d deposits within a few weeks, but did not affect microcirculation inflammation and intragraft gene expression patterns.^32^

Although survival differences were not significant in our primary study cohort, presumably due to the small sample size, the high-activity AP complotype turned out to be associated with adverse graft survival in a second large cohort of transplant recipients, not enriched for a specific type of rejection. The mechanisms behind this survival effect, however, need to be established in large studies powered to clarify the clinical impact of complement genetics in relation to posttransplant DSA and AMR occurrence. In this context, we want to point out that in the DSA-negative matched control group, we did not observe any graft survival differences, and, among a small subset of patients who neither had DSA before and after transplantation, all 3 graft losses occurred in patients without a high-risk complotype. Even though the results obtained in our primary cohort point to a role of glomerulitis/capillaritis triggered upon DSA-dependent complement activation, we are aware that outcome effects in our second cohort may not necessarily be related to antibody-mediated injury. Indeed, one may argue that survival differences reflect a multifaceted pathogenetic role of complement in different aspects of graft injury, including ischemia/reperfusion injury or T cell-dependent alloimmunity.^1^ The effect size in the second cohort was rather small, which may be explained by a contribution of complement-independent injury mechanisms in AMR, such as DSA/Fc gamma receptor-mediated natural killer cell activation or direct effects on the transplant endothelium mediated via HLA signaling.^33,34^ Another point is that common variants in complement genes only have a small effect on overall complement functionality compared with rare variants that are associated with complement-mediated disease. Consequently, one may expect that also low-activity variants allow for certain levels of complement activation that may trigger significant injury.

Interpreting our study results, we are aware of the limitation of sample size, which may have precluded detection of other subtle outcome differences in the primary study cohort. However, in this respect, we want to point out that a systematic prospective screening of >700 prevalent recipients was necessary to identify an untreated cohort of DSA-positive patients to specifically address the question whether and to which extent complement genotypes contribute to DSA pathogenicity. Another limitation was the lack of detailed data on DSA status and rejection occurrence in the second large cohort, which precluded additional subanalyses of survival differences in relation to DSA status or AMR. Finally, uniform ethnic demographics of our studied cohorts (the majority of included patients were Caucasian) may potentially limit generalizability of results. Indeed, previous studies have shown that distributions of C4 copy numbers markedly differed between ethnicities, with higher gene numbers of total C4 and associated variants among East-Asians.^35^ In addition, also frequencies of studied C3, fH and fB alleles were shown to substantially differ between different populations, including individuals of Caucasian, Asian, and/or African descent.^36-38^

In conclusion, the results of our study suggest that the intrinsic strength of the AP, maybe in part due to enhanced CP amplification, may determine the extent of microcirculation inflammation in DSA-positive patients. Preliminary results obtained in a large unselected transplant cohort suggest that a high-activity AP complotype may influence the long-term fate of kidney allografts. Future studies, however, will be needed to clarify the molecular mechanisms behind this possible outcome effect.

## Supplementary Material


