# Supplementary material for: High-activity Classical and Alternative Complement Pathway Genotypes—Association With Donor-specific Antibody-triggered Injury and Renal Allograft Survival
Source: Transplant Direct. 2020 Feb 10;6(3):e534. doi: 10.1097/TXD.0000000000000978 (PMC7056277; doi:10.1097/TXD.0000000000000978)
Supplement: Supplementary file 1 [file txd-6-e534-s001.pdf]

High-Activity Classical and Alternative Complement Pathway Genotypes – Association with Donor-Specific Antibody-Triggered Injury and Renal Allograft Survival

Blanka Mező, Roman Reindl-Schwaighofer, Farsad Eskandary, Andreas Heinzel, Markus Wahrmann, Konstantin Doberer, Andreas Heilos, Gregor Bond, Johannes Kläger, Nicolas Kozakowski, Helmuth Haslacher, Rainer Oberbauer, Ondřej Viklický, Petra Hrubá, Philip F. Halloran, Krisztina Rusai, Zoltán Prohászka, and Georg A. Böhmig

Supplementary Material

Table S1……………………………………………………………………………..………………..…**Page 2**
Table S2……………………………………………………………………………..………………..…**Page 3**
Table S3……………………………………………………………………………..………………..…**Page 4**
Table S4……………………………………………………………………………..………………..…**Page 5**
Table S5……………………………………………………………………………..…………………**Page 6 and 7**
Table S6……………………………………………………………………………..…………………**Page 8**
Table S7……………………………………………………………………………..…………………**Page 9 and 10**
Table S8……………………………………………………………………………..…………………**Page 11**

Table S1. Baseline characteristics – DSA-positive study patients versus DSA-negative matched control patients

Parameters	DSA+ (n=83)	DSA- (n=106)[a]	*P* value
**Variables recorded at the time of transplantation**			
Recipient age (years), median (IQR)	48 (36-54)	48 (38-56)	0.76
Female recipient sex, n (%)	37 (44.6)	45 (42.5)	0.88
Caucasian, n (%)	83 (100)	104 (98)[b]	0.51
Donor age (years), median (IQR)	46 (35-58)	48 (35-56)	0.95
Live donor, n (%)	13 (15.7)	18 (17.0)	0.85
ABO-incompatible allograft, n (%)	1 (1.2)	0 (0)	0.44
Recipient of a retransplant, n (%)	25 (30.1)	37 (34.9)	0.53
HLA mismatch in A, B and DR, median (IQR)	3 (2-4)	3 (2-4)	0.96
Current CDC panel reactivity ≥10%, n (%)[c]	14 (17.7)	16 (15.8)	0.84
Preformed anti-HLA DSA, n (%)[d]	24 (58.5)	19 (40.4)	0.13
Peritransplant immunoadsorption, n (%)[e]	25 (30.1)	19 (17.9)	0.05
**Variables recorded at the time of ABMR screening**			
Time to ABMR screening (years), median (IQR)	4.9 (1.7-13.1)	5.9 (2.9-10.6)	0.79
Recipient age (years), median (IQR)	55 (45-63)	55 (46-65)	0.84
Tacrolimus-based baseline immunosuppression	50 (60.2)	76 (71.7)	0.12
eGFR (ml/min/1.73 m^2), median (IQR)	54 (32-81)	63 (39-88)	0.088
Urinary protein/creatinine ratio (mg/g), median (IQR)	200 (79-488)	156 (83-453)	0.81

ABMR, antibody-mediated rejection; DSA, donor-specific antibody; CDC, complement-dependent cytotoxicity; eGFR, estimated glomerular filtration rate; IQR, interquartile range.

[a]The group of DSA-negative recipients was propensity score matched to DSA-positive study patients using female sex, recipient age at transplantation, urinary protein/creatinine ratio, prior transplantation, HLA mismatch and cytotoxic panel reactivity. For 1 (C4 genotyping) and 2 (complete evaluation of SNPs) cases, biological material was not sufficient for complete genotyping.

[b]Two recipients in the DSA-negative group were Asian.

[c]CDC panel reactivity was not recorded for 4 DSA-positive and 5 DSA-negative recipients.

[d]Pretransplant DSA data were available for 41 DSA-positive and 47 DSA-negative recipients (solid-phase HLA antibody screening on the waitlist was implemented at the Vienna transplant unit in July 2009).

[e]Sensitized patients (until 2009: ≥40% CDC-PRA; since 2009: preformed DSA) were subjected to peritransplant immunoadsorption.[34]

Table S2. Baseline demographics and patient characteristics – Vienna/Prague kidney transplant cohort[a]

Parameters	All patients (n=660)	High-activity C3/fB/fH complotype		P value
		yes (n=199)	no (n=461)	
Recipient age (years), median (IQR)	54 (42-62)	51 (41-60)	55 (44-63)	0.004
Female recipient sex, n (%)	249 (37.7)	66 (33.2)	183 (39.7)	0.11
Caucasian, n (%)	650 (98.5)	198 (99.5)	452 (98.0)	0.30
Glomerulonephritis as underlying renal disease, n (%)	230 (34.8)	80 (40.2)	150 (32.5)	0.06
Donor age (years), median (IQR)	53 (42-60)	54 (44-60)	52 (41-60)	0.27
Female donor sex, n (%)	289 (43.8)	92 (46.2)	197 (42.7)	0.41
Live donor, n (%)	94 (14.2)	35 (17.6)	59 (12.8)	0.11
Recipient of a retransplant, n (%)	75 (11.4)	29 (14.6)	46 (10)	0.09
Recipient presensitization[a]	244 (37)	80 (40.2)	164 (35.6)	0.26
HLA mismatch in A, B and DR, median (IQR)	3 (2-4)	3 (2-4)	3 (2-4)	0.99
Eplet mismatch, median (IQR)	42 (31-55)	43 (31-55)	41 (31-55)	0.76
Tacrolimus-based baseline immunosuppression	558 (84.5)	170 (85.4)	388 (84.2)	0.68
Induction with a depleting antibody	173 (26.2)	62 (31.2)	111 (24.1)	0.06

fB, complement factor B; fH, complement factor H; IQR, interquartile range.
[a]Donor-specific antibodies at the time of transplantation or cytotoxic panel reactivity >10 %.

Table S3. Primer and probe sequences for analysis of C4A and C4B CNV.

**Primer name**	**Sequence 5'-3'**
C4_Forward	GCAGGAGACATCTAACTGGCTTCT
C4_Reverse	CCGGACCTGCATGCTCCT
**Probe name[a]**	
C4A	ACCCCTGTCCAGTGTTAG
C4B	ACCTCTCTCCAGTGATAC

CNV, copy number variation.
[a]Underlined letters show the differences of the probes that distinguish the C4A and C4B genes.

Table S4. Primer sequences and PCR conditions used to determine $C3_{R102G}$ and fB_{R32Q} SNPs.

Primer name	Sequence 5'-3'	PCR conditions
C3_Forward[a]	AGTTGCTGACGCTGGTTGGA	95°C-15 min<br>94°C-20 sec, 57°C-10 sec, 72°C-110 sec (35 cycles)<br>72°C-10 min
C3_Reverse[a]	GCTTGTGGTTGACGGTGAAGAT	
fB_Forward[a]	GGGAAAGTGATGTGGGTAGGAC	95°C-15 min<br>95°C-15 sec, 60,9°C-30 sec, 72°C-30 sec (35 cycles)<br>72°C-10 min
fB_Reverse[a]	GCACAGGGTACGGGTAGAAG	
fB_e1Forward[b]	TCACATGGAATTTCCCAGTTATG	95°C-5 min<br>95°C-15 sec, 59°C-15 sec, 72°C-150 sec (35 cycles)<br>72°C-10 min
fB_e3Reverse2[b]	CAGTGGTAGGTGACGCTGTCT	
fB_e2Reverse[b]	TGTCACCCTGCCTAGTCTCATC	96°C-1 min<br>96°C-10 sec, 56°C-10 sec, 60°C-4 min (25 cycles)

fB, complement factor B; SNP, single nucleotide polymorphism.
[a]Primers used for RFLP-PCR reaction.
[b]Primers used for PCR reaction and sequencing.

Table S5. Genotype distributions and allele frequencies in DSA-positive recipients and matched DSA-negative controls.

Gene; polymorphism	DSA+ study recipients (N=83)		DSA- control subjects (N=106)		*P* value (study vs. control subjects)
	Observed (%)	Expected (%)[a]	Observed (%)	Expected (%)[a]	
**C3**					
rs2230199 (c.304C>G; p.R102G; C3S and C3F)					
**Genotype[b], n (%)**					
**G**/**G** (**102G**/**102G**)	6 (7.2)	3.3 (4.0)	6 (5.8)	2.6 (2.5)	0.62
**G**/C (**102G**/102R)	21 (25.3)	26.4 (31.8)	21 (20.2)	27.8 (26.7)	
C/C (102R/102R)	56 (67.5)	53.3 (64.2)	77 (74.0)	73.6 (70.8)	
***P* value (observed vs. expected)**	0.061		0.013		
**Allelic frequency[b], n (%)**					
**G** (**102G**)	33 (19.9)		33 (15.9)		
C (102R)	133 (80.1)		175 (84.1)		
**fB**					
rs641153 (c.95G>A; FB R32Q)					
**Genotype[b], n (%)**					
**G**/**G** (**32R**/**32R**)	65 (78.3)	66.0 (79.5)	91 (87.5)	91.4 (87.9)	
**G**/A (**32R**/32Q)	18 (21.7)	16.0 (19.3)	13 (12.5)	12.2 (11.7)	0.093
A/A (32Q/32Q)	0 (0.0)	1.0 (1.2)	0 (0.0)	0.4 (0.4)	
***P* value (observed vs. expected)**	0.268		0.497		
**Allelic frequency[b], n (%)**					
**G** (**32R**)	148 (89.2)		195 (93.8)		
A (32Q)	18 (10.8)		13 (6.3)		
**fH**					
rs800292 (c.184G>A; FH V62I)					
**Genotype[b], n (%)**					
**G**/**G** (**62V**/**62V**)	50 (60.2)	50.1 (60.4)	69 (66.3)	70.3 (67.6)	
**G**/A (**62V**/62I)	29 (34.9)	28.8 (34.7)	33 (31.7)	30.4 (29.2)	
A/A (62I/62I)	4 (4.8)	4.1 (4.9)	2 (1.9)	3.3 (3.2)	0.45
***P* value (observed vs. expected)**	0.938		0.387		

**Allelic frequency[b], n (%)**		
**G** (**62V**)	129 (77.7)	171 (82.2)
A (62I)	37 (22.3)	37 (17.8)

DSA, donor-specific antibody; fB, complement factor B; fH, complement factor H.
[a]Expected genotype frequencies at Hardy Weinberg equilibrium were calculated from allele frequencies in study and control subjects.
[b]Risk variants are marked with bold font.

Table S6. Complement gene variants in DSA-positive recipients in relation to complement profile, biopsy results and survival.

	**C3** rs2230199 (c.304C>G; p.R102G)				**fB** rs641153 (c.95G>A; p.R32Q)				**fH** rs800292 (c.184G>A; p.V62I)			
	**G/G** (**102G/102G**) (n=6)	**G**/C (**102G**/102R) (n=21)	C/C (102R/102R) (n=56)	***P* value**	**G/G** (**32R/32R**) (n=65)	**G**/A (**32R**/32Q) (n=18)	A/A (32Q/32Q) (n=0)	***P* value**	**G/G** (**62V/62V**) (n=50)	**G**/A (**62V**/62I) (n=29)	A/A (62V/62I) (n=4)	***P* value**
**Blood complement profile, median (IQR)**												
C3, g/L	1.24 (1.09-1.40)	1.20 (1.08-1.46)	1.43 (1.21-1.61)	0.02	1.38 (1.20-1.54)	1.39 (1.09-1.65)	-	0.847	1.38 (1.20-1.55)	1.38 (1.19-1.58)	1.43 (1.12-1.75)	0.78
C4, g/L	0.33 (0.29-0.41)	0.30 (0.25-0.37)	0.35 (0.26-0.42)	0.25	0.33 (0.26-0.39)	0.38 (0.30-0.45)	-	0.093	0.35 (0.26-0.41)	0.31 (0.26-0.41)	0.34 (0.29-0.53)	0.73
CH50, U/mL	51 (49-55)	59 (50-70)	62 (57-70)	0.035	62 (51-72)	62 (59-67)		0.934	61 (52-67)	63 (53-82)	75 (63-86)	0.042
%AP activity	114 (112-117)	103 (92-110)	110 (102-115)	0.014	110 (102-114)	109 (100-115)	-	0.711	110 (100-115)	109 (100-114)	118 (102-130)	0.27
**Biopsy results, n (%)**												
ABMR	4 (66.7)	15 (71.4)	28 (50.0)	0.21	37 (56.9)	10 (55.6)	-	0.563	26 (52.0)	18 (62.1)	3 (75.0)	0.51
C4d+ABMR	2 (33.3)	6 (28.6)	13 (23.2)	0.797	17 (26.2)	4 (22.2)	-	0.499	13 (26.0)	8 (27.6)	0 (0)	0.49
**5-year survival rates, %**												
Death-censored	65	80	84	0.493	79	89	-	0.468	87	71	100	0.18
Patient survival	100	85	82	0.518	82	88	-	0.482	85	83	75	0.87

ABMR, antibody-mediated rejection; fB, complement factor B; fH, complement factor H.

Table S7. Genotype distributions and allele frequencies in DSA-positive recipients and the Vienna/Prague transplant cohort.

Gene; polymorphism	DSA+ study recipients (N=83)		Vienna/Prague cohort (N=660)		*P* value (DSA-positive vs. Vienna/Prague cohort)
	Observed (%)	Expected (%)[a]	Observed (%)	Expected (%)[a]	
**C3**					
rs2230199 (c.304C>G; p.R102G)					
**Genotype, n (%)**					
**G**/**G** (102G/102G)	6 (7.2)	3.3 (4.0)	16 (2.4)	18.5 (2.8)	
**G**/C (102R/102G)	21 (25.3)	26.4 (31.8)	189 (28.6)	184 (27.9)	0.048
C/C (102R/102R)	56 (67.5)	53.3 (64.2)	455 (68.9)	457.5 (69.3)	
***P* value (observed vs. expected)**	0.061		0.485		
**Allelic frequency, n (%)**					
**G** (102G)	33 (19.9)		221 (16.7)		
C (102R)	133 (80.1)		1099 (83.3)		
**fB**					
rs641153 (c.95G>A; FB R32Q)					
**Genotype, n (%)**					
**G**/**G** (32R/32R)	65 (78.3)	66.0 (79.5)	563 (85.3)	561.9 (85.1)	
**G**/A (32R/32Q)	18 (21.7)	16.0 (19.3)	92 (13.9)	94.1 (14.3)	0.19
A/A (32Q/32Q)	0 (0.0)	1.0 (1.2)	5 (0.8)	3.9 (0.6)	
***P* value (observed vs. expected)**	0.268		0.563		
**Allelic frequency, n (%)**					
**G** (32R)	148 (89.2)		1218 (92.3)		
A (32Q)	18 (10.8)		102 (7.7)		
**fH**					
rs800292 (c.184G>A; FH V62I)					
**Genotype, n (%)**					
**G**/**G** (62V/62V)	50 (60.2)	50.1 (60.4)	415 (62.9)	409.7 (62.1)	
**G**/A (62V/62I)	29 (34.9)	28.8 (34.7)	210 (31.8)	220.6 (33.4)	0.84
A/A (62I/62I)	4 (4.8)	4.1 (4.9)	35 (5.3)	29.7 (4.5)	
***P* value (observed vs. expected)**	0.938		0.217		

**Allelic frequency, n (%)**		
**G** (62V)	129 (77.7)	1040 (78.8)
A (62I)	37 (22.3)	280 (21.2)

DSA, donor-specific antibody; fB, complement factor B; fH, complement factor H.
[a]Expected genotype frequencies at Hardy Weinberg equilibrium were calculated from allele frequencies.

Table S8. High-activity C3/fB/fH complotype and death-censored graft survival in the Vienna/Prague kidney transplant cohort - multivariate Cox regression analysis.

**Variable**	**Hazard ratio**	**95% confidence interval**	**p-value**
High-activity C3/fB/fH complotype, yes vs. no	1.55	1.04 – 2.32	0.031
Recipient age >65 years, yes vs. no	1.46	0.85 – 2.51	0.17
Female recipient sex, yes vs. no	1.17	0.79 – 1.75	0.44
Glomerulonephritis as primary renal disease, yes vs. no	0.70	0.45 – 1.08	0.11
Donor age, per year	1.02	1.01 – 1.04	0.004
Female donor sex, yes vs. no	1.66	1.11 – 2.49	0.015
Live donor, yes vs. no	1.02	0.60 – 1.74	0.94
Retransplant, yes vs. no	3.17	1.90 – 5.28	<0.001
HLA eplet mismatch, per increase in mismatch score	1.01	1.00 – 1.02	0.013
Tacrolimus-based baseline immunosuppression, yes vs. no	1.10	0.65 – 1.89	0.72
Induction with a depleting antibody, yes vs. no	0.76	0.47 – 1.23	0.26

fB, complement factor B; fH, complement factor H.
